# A screening question to assess risk of using antibiotics without a prescription: a diagnostic study

**DOI:** 10.1186/s12875-025-02811-3

**Published:** 2025-04-15

**Authors:** Ashley Collazo, Eva Amenta, Kiara Olmeda, Marissa Valentine-King, Lindsey Laytner, Azalia Mancera, Roger Zoorob, Michael K. Paasche-Orlow, Richard L. Street Jr, Barbara W. Trautner, Larissa Grigoryan

**Affiliations:** 1 Department of Family & Community Medicine, Baylor College of Medicine, 3701 Kirby Dr., Suite 655, Houston, TX 77098 USA; 2 Department of Medicine, Section of Infectious Diseases, Baylor College of Medicine, 2002 Holcombe Boulevard, Houston, TX 77030 USA; 3 Center for Innovations in Quality, Effectiveness and Safety (IQuESt), Michael E. DeBakey Veterans Affairs Medical Center, 2450 Holcombe Blvd Suite 01Y, Houston, TX 77021 USA; 4 Department of Medicine, Tufts Medical Center, 260 Tremont Street, Biewend Building, Floor 6, Boston, MA 02116 USA; 5 Department of Communication, Texas A&M University, College Station, TX 77843 USA; 6 Department of Medicine, Section of Health Services Research, Baylor College of Medicine, One Baylor Plaza, BCM 288, Houston, TX 77030 USA

**Keywords:** Antibiotic stewardship, Primary health care, Bacterial drug resistance, Anti-bacterial agents, Predictive value of tests

## Abstract

**Objectives:**

Non-prescription antibiotic use (using antibiotics without medical advice) is potentially unsafe and promotes antimicrobial resistance. We studied predictors of prior non-prescription use and whether screening for prior non-prescription antibiotic use predicted intention of future non-prescription antibiotic use.

**Methods:**

The survey was performed from January 2020 - June 2021 in six public primary care clinics and two private emergency departments. Prior non-prescription users were respondents who reported taking oral antibiotics for symptoms without contacting a clinician. Intended use was defined by answering yes to the question, “*would you use antibiotics without contacting a doctor/nurse/dentist/clinic*.” We examined predictors for prior non-prescription use. We also calculated the sensitivity, specificity, and positive and negative predictive value (PPV, NPV) of (a) any prior non-prescription antibiotic use and (b) prior use in the past 12 months - for future intended non-prescription use.

**Results:**

Of 564 survey respondents, 246 (43.6%) reported non-prescription use; 91 (37.0%) of these respondents, 16.1% overall, reported doing so in the past 12 months. Approximately 63% of non-prescription antibiotic use was in those with a previous prescription of the same antibiotic for similar symptoms/illnesses. The screening characteristics of non-prescription use in the past 12 months to identify intention to use of antibiotics without a prescription in the future were: sensitivity 75.9% (95% CI: 65.3–84.6), specificity 91.4% (95% CI: 87.8–94.2), Bayes’ PPV 74.5% (95% CI: 66.7–80.9), and Bayes’ NPV 93.7% (95% CI: 90.5–96.1).

**Conclusions:**

This study proposed a method to screen for future use of non-prescription antibiotics, which may have implications on antimicrobial stewardship efforts in primary care settings.

## Background

Taking antibiotics without a prescription (non-prescription use) incurs risks to patients and public health [1]. Risks of non-prescription antibiotic use include potential adverse drug reactions, drug interactions, diagnostic delays, *Clostridioides difficile* infections, and disruption of the microbiome [1]. Further, the practice of taking antibiotics outside of a healthcare relationship adds to the global risk of antimicrobial resistance, which accounted for 4.95 million deaths globally in 2019 [1–4].

Non-prescription antibiotic use includes taking antibiotics from prior unfinished prescriptions, sharing “leftover” antibiotics amongst friends or family members, and obtaining antibiotics from flea markets, ethnic markets (e.g., yerberias), the internet, or outside the United States [5, 6]. A review of the recent literature surrounding non-prescription antibiotic use in the United States (US) found between 20 and 45% [7–9] of surveyed individuals used non-prescription antibiotics, and 25% reported an intention to use antibiotics without a prescription [10]. Identifying individuals at risk for non-prescription antibiotic use is key to preventing this unsafe practice. Previous studies found a strong association between prior non-prescription antibiotic use (prior use) and professed intention to use non-prescription antibiotics in the future (intended use) [8, 10, 11]. In a 2006 multi-country European study, non-prescription use in the preceding 12 months was strongly associated with intended use of non-prescription antibiotics [10].

We studied the diagnostic properties of a brief screening question inquiring about non-prescription antibiotic use at two time frames for non-prescription antibiotic use (ever or in the past 12 months) to identify patients with intention to use antibiotics without medical guidance in the future. These two time frames for prior non-prescription use (ever or previous 12 months) were chosen to compare their diagnostic accuracies. We also determined the prevalence and predictors of prior non-prescription use, as well as the symptoms/illnesses prompting individuals to use non-prescription antibiotics and the sources of antibiotic acquisition.

## Methods

The survey was conducted between January 2020 and June 2021 among adult patients in waiting rooms at six public clinics (half continuity, half same day) and two private emergency departments that serve ethnically and socioeconomically diverse patients in Houston, Texas, and its more affluent suburbs. Our multistage sampling design was selected to ensure economic, educational, racial, ethnic, and linguistic diversity. Participants were recruited by flyer upon check in at participating locations. The flyer described the study and provided instructions on contacting the research coordinator if interested in participating in the survey interviews. The survey interviews were completed anonymously in the respondent’s preferred language (English or Spanish). Initially, interviews were conducted in person in the waiting areas by a research coordinator. However, from March 2020-May 2021, coordinators were excluded from waiting areas due to the 2019 coronavirus pandemic (COVID-19); therefore, these interviews were performed by phone. Informed consent was obtained from all participants in the study. Those unable to answer survey questions and who were not 18 years of age or older were excluded from the study. The survey instrument and additional details of the study design are published elsewhere [7]. The study was conducted in accordance with the Declaration of Helsinki and approved by the Baylor College of Medicine Institutional Review Board (protocol #: H-45709).

### Survey instrument

The survey asked respondents about habits surrounding antibiotics, including non-prescription antibiotic use and clinical reasons (i.e., symptoms or illness) for taking the antibiotic. Respondents were classified as non-prescription users if they reported having previously taken antibiotics without a prescription at any time. Non-prescription antibiotic use was further classified as any use in the past (ever) or use within the past 12 months by responding to the prompt, *“When was your most recent experience with taking an antibiotic without contacting a doctor/dentist/nurse?”* Intended use (primary outcome) was defined as professed intention to take antibiotics without a prescription in the future, and was measured by the question, *“In general*,* would you use antibiotics without contacting a doctor/nurse/dentist/clinic?”* Answers of “yes” or “maybe” were coded as intended use as this response indicated the participant was open to non-prescription antibiotic use. Responses “no” and “I don’t know” were coded as no intended use. This categorization has been used in our previous studies on non-prescription use [8, 12, 13]. Only five survey participants reported “I don’t know” and thus combined with “no” responses.

In those who reported non-prescription use, we asked them to specify the symptoms prompting them to use non-prescription antibiotics, the sources of acquisition and the types of antibiotics used. We also asked whether the same antibiotic was previously prescribed for the same reason (symptom/illness). Symptoms were grouped into six broad categories: sore throat, cold/flu/upper respiratory infection (URI), dental symptoms, skin/wound infections, sinus infection, and other. The other category included a wide variety of reported symptoms with very low prevalence or instances when a participant was unable to recall a symptom. The source of antibiotics was separated into four categories: abroad, family or friends, leftover prescription antibiotics, and other. Other sources included non-clinical locations like markets, the internet, or places of work (i.e., veterinary sources) or when the patient was unable to recall where they acquired the antibiotic. To assist survey participants with identifying the type of antibiotic taken, the research coordinator provided the participant with a list of commonly used antibiotics in the U.S. and Latin American countries (brand and generic names) with photos for reference. For those participants who reported more than one instance of taking a non-prescription antibiotic, only the first instance was explored with further questions as to the antibiotic source and name.

We also collected the following participant data: age, gender, race, ethnicity, education level, household income, health insurance status, health literacy, overall health status, and country of birth. The parts of the survey that did not have a validated Spanish version were translated using a combination of committee translation and standard back-translation strategies to achieve semantic equivalence.

The survey was designed with input from a community advisory board composed of six ethnically diverse patient representatives from participating clinics. In response to input from the community advisory board and team members with sociocultural and health literacy expertise, items were modified for reading level, clarity, and cultural appropriateness.

### Data analysis

Descriptive statistics were calculated on all study variables. To evaluate test metrics of the screening question, we considered the screening question (prior non-prescription use) as the ‘test’ and intended antibiotic use (the outcome) as the ‘gold standard.’ We studied the test characteristics of the screening question for two time frames: (1) any prior non-prescription antibiotic use (ever) and (2) non-prescription use in the past 12 months. We calculated test metrics for each version of the screening question using sensitivity, specificity, Bayes’ positive predictive value, and Bayes’ negative predictive values [14, 15].

In the context of our study, sensitivity is the screening question’s ability to detect patients who intend to use antibiotics without a prescription, while specificity is the ability of the screening question to detect those who do not intend to use non-prescription antibiotics. The PPV and NPV represent the probability of the screening question to accurately classify those who intend and do not intend to use non-prescription antibiotics, respectively. We used Bayes’ version of PPV and NPV to incorporate the prevalence of intended non-prescription antibiotic use (or pre-test probability of non-prescription antibiotic use) to interpret the PPV more accurately.

The relationships between any prior use and respondent sociodemographic characteristics were evaluated using univariate and multivariate logistic regression with adjustment for sociodemographic factors (i.e., age, race/ethnicity, gender, and health literacy [16, 17]). All analyses were done using R Studio Version 2023.12.1 + 402 [18].

## Results

### Patient characteristics

We surveyed 564 patients with a median age of 51 years (Interquartile Range = 41–60) (Table 1). Nearly three-quarters of participants (*n* = 409, 72.5%) were surveyed in a public clinic, while 155 (27.5%) were surveyed in a private emergency department. Most respondents were either Hispanic or Latino (46.6%) or African American (33%), females (72.2%), and born in the U.S. (63.8%). Most respondents had Medicaid or the county-assisted financial assistance program, allowing access to publicly funded clinics at either very low or no cost.

**Table 1:** Sociodemographic characteristics of respondents (*n* = 564)

Characteristic	Value	Any Prior Non-Prescription Antibiotic Use			
		Yes *N* = 246	No *N* = 318	Unadjusted OR (95% CI)	Adjusted^f^ OR (95% CI)
Median age (year) (IQR)^a^	51 (41–60)	48.7 (11.9)	50.5 (14.8)	0.99 (0.98-1.00)	…
No. (%) of female respondents	407 (72.2)	177 (72.0)	230 (72.3)	0.97 (0.67–1.41)	…
No. (%) of respondents of race and ethnicity					
African American or Black	186 (33.0)	80 (32.5)	106 (33.3)	1 (reference)	…
Hispanic or Latina/Latino	263 (46.6)	123 (50.0)	140 (44.0)	0.86 (0.59–1.25)	…
Other^a^	26 (4.6)	6 (2.4)	20 (6.3)	0.81 (0.50–1.31)	…
Non-Hispanic White	89 (15.8)	37 (15.0)	52 (16.4)	0.34 (0.12–0.83)	…
No. (%) of respondents with education level					
Less than high school	92 (16.3)	37 (15.0)	55 (17.3)	1 (reference)	1 (reference)
High school or GED	225 (39.9)	95 (38.6)	130 (40.9)	1.09 (0.66–1.79)	1.23 (0.72–2.12)
Some college and above	247 (43.8)	114 (46.3)	133 (41.8)	1.27 (0.79–2.08)	1.54 (0.86–2.76)
No. (%) of respondents with insurance status					
Private, Medicare	207 (36.7)	75 (30.5)	132 (41.5)	1 (reference)	1 (reference)
Medicaid, Harris Health Financial Assistance program^b^	319 (56.6)	156 (63.4)	163 (51.3)	1.68 (1.18–2.42)	1.56 (1.07–2.27)
Self-pay	38 (6.7)	15 (6.1)	23 (7.2)	1.15 (0.56–2.32)	1.00 (0.47–2.08)
No. (%) of patients vising public vs. private healthcare system					
Private	155 (27.5)	48 (19.5)	107 (66.4)	1 (reference)	1 (reference)
Public	409 (72.5)	198 (80.5)	211 (33.6)	0.48 (0.32–0.70)	0.40 (0.25–0.62)
No. of respondents with income/total no. of respondents (%)					
<$20,000	254 (45.0)	125 (50.8)	129 (40.6)	1 (reference)	1 (reference)
≥$20,000 but <$40,000	98 (17.4)	43 (17.5)	55 (17.3)	0.81 (0.50–1.29)	0.75 (0.46–1.21)
≥$40,000 but <$60,000	34 (6.0)	13 (5.3)	21 (6.6)	0.64 (0.30–1.32)	0.54 (0.25–1.15)
≥$60,000 but <$100,000	23 (4.1)	10 (4.1)	13 (4.1)	0.79 (0.33–1.87)	0.72 (0.29–1.74)
<$100,000	37 (6.6)	10 (4.1)	27 (8.5)	0.38 (0.17–0.80)	0.36 (0.15–0.81)
Do not know/prefer not to say	118 (20.9)	45 (18.3)	73 (23.0)	0.64 (0.41–0.99)	0.60 (0.38–0.94)
No. (%) of questionnaires completed in Spanish	155 (27.5)	67 (27.2)	88 (27.7)	0.98 (0.67–1.42)	0.75 (0.45–1.25)
No. (%) of respondents born in the United States/Other					
United States	360 (63.8)	160 (65.0)	200 (62.9)	1 (reference)	1 (reference)
Other^c^	204 (36.2)	86 (35.0)	99 (37.1)	0.91 (0.64–1.29)	0.73 (0.45–1.17)
Median years lived in United States for the respondents born in other countries (year) (IQR)	23 (17–31)	23.7 (12.9)	25.2 (11.7)	1.00 (0.99-1.00)	1.01 (0.99–1.02)
No. (%) of respondents reporting use of non-prescription antibiotics					
Prior non-prescription use Non-prescription use in the past 12 mo.	246 (43.6) 91 (16.1)	…	…	…	…
No. (%) of respondents reporting intention to use non-prescription antibiotics					
Overall Non-prescription use in the past 12 mo.	140 (24.8) 63 (11.2)	120 (48.8)	20 (6.3)	…	…
Overall health status mean (SD)^d^	3.3 (1.1)	3.4 (1.1)	3.2 (1.1)	1.18 (1.00-1.38)	1.20 (1.02–1.41)
No. (%) Health Literacy^e^					
Adequate Health Literacy	410 (72.7)	172 (69.9)	219 (68.9)	1 (reference)	…
Inadequate Health Literacy	154 (27.3)	74 (30.1)	99 (31.1)	0.95 (0.66–1.36)	…

^a^Includes: 10 Asian, 10 self-reported as multiracial or multiethnic, 4 American Indian, 2 declined

^b^County financial assistance program includes those who have benefits from the county allowing access to public clinic providers at either very low cost or no cost

^c^Includes: 1 Africa, 1 Barbados, 1 Canada, 1 Columbia, 1 Costa Rica, 6 Cuba, 1 Declined to provide, 1 Dominican Republic, 14 El Salvador, 6 Guatemala, 15 Honduras, 1 India, 2 Iran, 1 Jamaica, 2 Jordan, 2 Korea, 1 Lebanon, 131 Mexico, 2 Nicaragua, 3 Nigeria, 2 Pakistan, 1 Panama, 1 Peru, 1 Philippines, 1 Saudi Arabia, 3 Venezuela, 2 Vietnam (countries are listed in alphabetical order*)*

^d^Score ranges from 1 to 5 (excellent = 1; poor = 5), with lower scores being more favorable

^e^Calculated using the three questions from the Brief Health literacy Screen measure [16, 17]

^f^Adjusted for age, race/ethnicity, gender, and health literacy

Prior non-prescription antibiotic use was reported by 246 of 564 (43.6%) individuals, with 91 (16.1%) reporting use within the last 12 months. For our outcome, 140 of 564 participants (24.8%) intended to use non-prescription antibiotics.

### Sensitivity and specificity

The sensitivity of the screening questions for detecting intended non-prescription antibiotic use was 85.7% (95% confidence interval (CI): 78.8–91.1) when screening for any prior non-prescription use and 75.9% (95% CI: 65.3–84.6) when asking about non-prescription use in the past 12 months. The specificity of the screening questions for identifying those not at risk for non-prescription use was 70.3% (95% CI: 65.7–74.6) when screening for any prior non-prescription antibiotic use and 91.4% (95% CI: 87.8–94.2) when screening for non-prescription use in the past 12 months (Table 2).

**Table 2:** Diagnostic accuracy of the screening questions on prior non-prescription antibiotic use to identify intended users

Screening question	Sensitivity (95% CI)	Specificity (95% CI)	PPV (95% CI)	NPV (95% CI)	Bayes PPV^b^ (95% CI)	Bayes NPV^b^ (95% CI)
Any prior use of antibiotics without a prescription (*n* = 564)	85.7% (78.8–91.1)	70.3% (65.7–74.6)	48.8% (42.4–55.2)	93.7% (90.5–96.1)	48.8% (45.1–52.5)	93.7% (92.9–94.6)
Prior use of non-prescription antibiotics in the last 12 months (*n* = 409)^a^	75.9% (65.3–84.6)	91.4% (87.8–94.2)	69.2% (58.7–78.5)	93.7% (90.5–96.1)	74.5% (67.7–81.2)	92.0% (90.4–93.6)

^a^Sample excludes population whose most recent non-prescription antibiotic use was more than 12 months ago

^b^The prevalence of intended non-prescription antibiotic use (24.8%) was used in this calculation

### Positive and negative predictive values

For the screening question on any prior non-prescription antibiotic use, Bayes’ PPV and NPV were 48.8% (95% CI: 45.1–52.5) and 93.7% (95% CI: 92.9–94.6), respectively **(**Table 2). For the screening question on non-prescription antibiotic use in the past 12 months, Bayes’ PPV and NPV were 74.5% (95% CI: 67.7–81.2) and 92.0% (95% CI: 90.4–93.6), respectively. Bayes’ PPV and NPV for both any prior non-prescription antibiotic use and use in the last 12 months were calculated using the prevalence of intended non-prescription antibiotic use (24.8%).

### Symptoms/illnesses reported for non-prescription antibiotic use and sources of acquisition

**Fig. 1:**
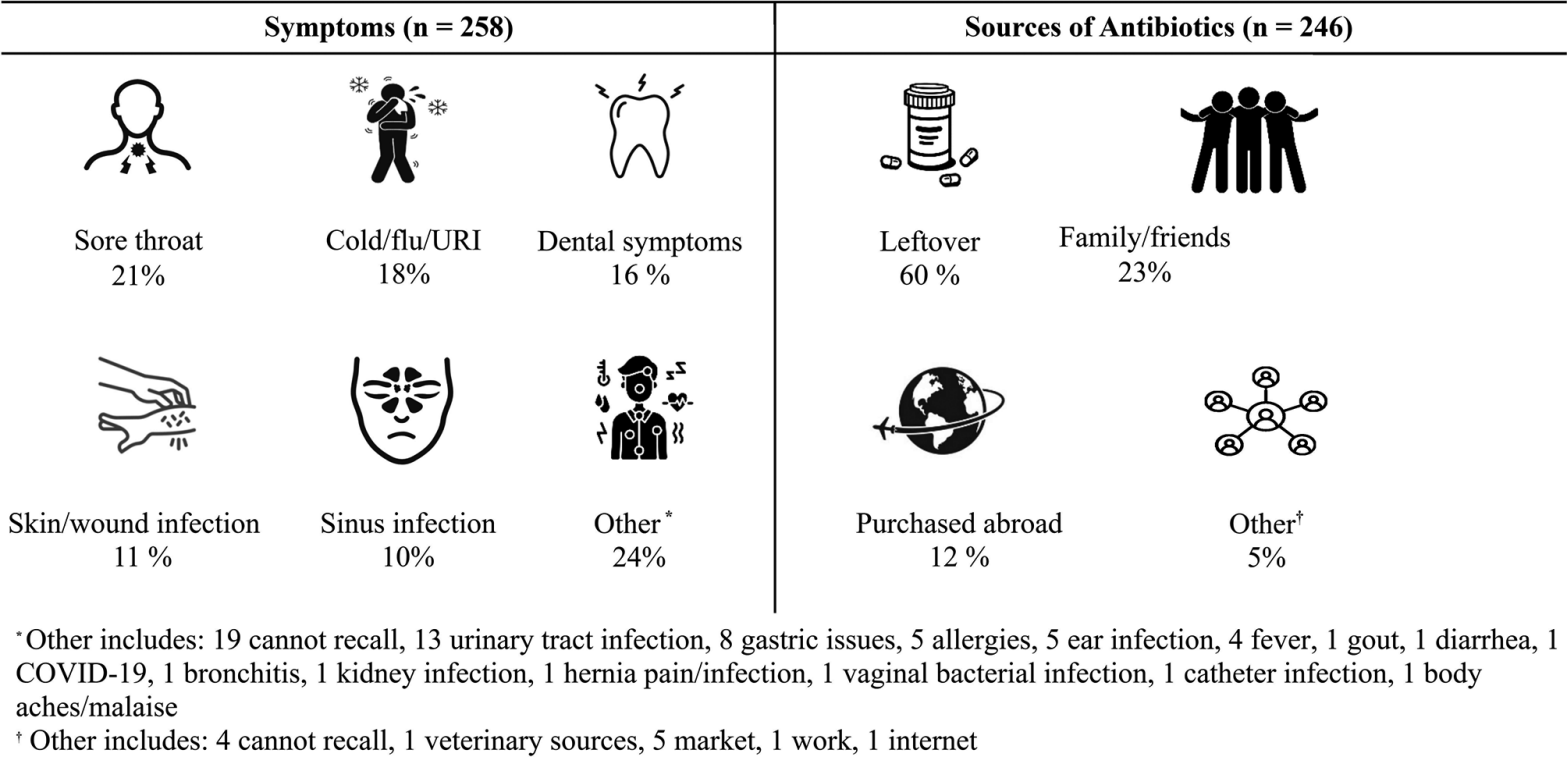
Symptoms and Sources of Non-Prescription Antibiotic Use

There were 258 reported symptoms/illnesses that led the 246 respondents to use non-prescription antibiotics, as 12 participants reported more than one symptom. Of all symptoms/illnesses reported for non-prescription use, the most common were sore throat (20.5%), cold/flu/URI (17.8%), and dental symptoms (16.3%). Other reported symptoms/illnesses included urinary tract infections, gastric issues, and allergies. (Fig. 1). The most common sources for non-prescription antibiotics were leftover prescribed antibiotics (60.2%), antibiotics from friends or relatives (22.8%), or antibiotics purchased abroad (12.2%). Other sources reported were the local market, veterinary sources, work, and the internet. (Fig. 1).

### Type of non-prescription antibiotic and previous prescription of antibiotic for the same symptoms

The most common non-prescription antibiotic used was amoxicillin at 37.4% followed by penicillin at 6.9%. Among the other non-prescription antibiotics reported, there were trimethoprim/sulfamethoxazole, ciprofloxacin, and tetracycline. (Table 3). Of the 246 participants reporting non-prescription antibiotic use, 62.6% reported that the antibiotic they took had been previously prescribed for the same symptom/illness. (Table 3)

**Table 3:** Type of non-prescription antibiotic and previous prescription of antibiotic for the same symptoms (*n* = 256)

Survey response	*n* (%)
Type of Non-Prescription Antibiotic Used	
Amoxicillin	92 (37.4)
Penicillin	17 (6.9)
Ampicillin	13 (5.3)
Cephalexin	8 (3.3)
Azithromycin	7 (2.8)
Trimethoprim/sulfamethoxazole	7 (2.8)
Ciprofloxacin	4 (1.6)
Tetracycline	3 (1.2)
Clindamycin	2 (0.8)
Nitrofurantoin	2 (0.8)
Doxycycline	1 (0.4)
Lincomycin	1 (0.4)
Metronidazole	1 (0.4)
Cannot Recall	88 (35.8)
Previously prescribed^a^	
Yes	154 (62.6)
No	77 (31.3)
Cannot Recall	15 (6.1)

^a^Same antibiotic had been previously prescribed to the survey respondent for similar symptoms as those that prompted the non-prescription antibiotic use

### Predictors of prior non-prescription antibiotic use

Type of insurance (*P* = 0.049), healthcare system used (*P* < 0.001), and overall health status (*P* = 0.029) were significantly associated with prior non-prescription use after adjustment for age, gender, race/ethnicity, and health literacy. Those who have Medicaid or use the county financial assistance program were more likely to report prior non-prescription use than those with private or Medicare insurance (adjusted odds ratio (aOR) 1.56, 95% CI: 1.07–2.27). Participants receiving care in private clinics were less likely to use non-prescription antibiotics than participants that receive care in public clinics (aOR = 0.40, 95% CI: 0.25–0.62). Those who self-reported poorer health were more likely to use non-prescription antibiotics than those with better overall self-reported health (aOR = 1.20, 95% CI: 1.02–1.41). (Table 1)

## Discussion

Our survey found that nearly half (43.6%) of respondents had previously used antibiotics without a prescription. Most often, these prior users acquired antibiotics from leftover prescriptions (60.2%). Many prior users (62.6%) reported using non-prescription antibiotics for similar symptoms to what had previously been treated with antibiotics, with amoxicillin being the most commonly used non-prescription antibiotic (34.8%). Predictors associated with prior non-prescription antibiotic use were utilizing the public health care system, having Medicaid or the county-assisted financial assistance program, and self-reported poorer overall health.

The question screening for any prior non-prescription antibiotic use had high sensitivity (85.7%) in detecting future non-prescription antibiotic users. However, this question had a PPV capable of accurately predicting non-prescription use in less than half (48.8%) that screened positive. Screening for non-prescription antibiotic use in the past 12 months exhibited a sensitivity of 75.9% and PPV of 74.5%. Thus, screening for non-prescription antibiotic use in the past 12 months will potentially identify a substantial number of intended users and reduce time spent on those with no intention of non-prescription antibiotic use. Due to possible stigma surrounding the use of non-prescription antibiotics, asking patients about prior use may detect more non-prescription antibiotic users than asking about intention to use, therefore capturing more patients at risk for this practice.

The prevalence of prior non-prescription antibiotic use in our study population was 43.6% which fell in the range of the prevalence reported in other recent U.S. studies (20-45%) [7–9]. Our study had a lower prevalence of non-prescription antibiotic use than prevalence previously reported in US-Mexico border counties, presumably due to the ease of access to antibiotics in those locations [9, 19]. Leftover prescriptions are a major source of antibiotic self-medication worldwide, as evidenced by studies in the United States [5, 8, 9, 14, 19–21] and Europe [10, 11], with penicillin-based antibiotics being the most commonly used non-prescription antibiotics [9, 22].

This screening tool can serve multiple purposes. On a patient-provider level, this screening tool may have the potential to help providers quickly identify patients at risk and target education about the harms of non-prescription antibiotic use to those at the highest risk and for whom this topic is most salient. Focusing the providers’ efforts on the highest-risk individuals will likely maximize the benefits of these efforts. Acting at the patient-provider level is also ideal, as previous interventions in primary care clinics successfully reduced antibiotic use with written educational material in combination with patient-provider discussion about appropriate antibiotic use [23, 24]. At the community level, it could possibly be used in public health interventions to more accurately tailor interventions to those at highest risk for non-prescription antibiotic use.

At all levels, education is key in addressing non-prescription antibiotic use. The practice of using non-prescription antibiotics for self-limiting symptoms/illness can be addressed with an open patient-provider discussion explaining the lack of antibiotic impact on self-limited illnesses like acute lower respiratory tract infections [25] and education on symptom management [23]. One strategy to reduce non-prescription antibiotic use may be to reduce the availability of leftover antibiotics by prescribing the shortest possible duration of appropriate antibiotic courses. Another potential strategy is educating patients on the adverse effects of antibiotics [21]. For example, severe allergic reactions to penicillin and sulfa-based drugs as well as risks of nephrotoxicity with ciprofloxacin, trimethoprim/sulfamethoxazole, and expired tetracycline [26]. Of particular concern is over 62% of respondents reporting non-prescription antibiotic use said they took an antibiotic that had previously been prescribed for similar symptoms. This shows the initial decision of the provider to prescribe antibiotics may have a powerful influence on patients’ subsequent antibiotic choices outside the healthcare system.

To our knowledge, this is the first study to validate a screening question to predict the use of non-prescription antibiotics. We used Bayes’ PPV and NPV, which helps interpret our results in the context of intended use of non-prescription antibiotic prevalence and allows for generalizability to other populations with similar levels of non-prescription antibiotic. In terms of limitations, we surveyed individuals in healthcare settings, potentially underestimating rates of non-prescription use. Our survey was cross-sectional, meaning that both the exposure (prior non-prescription antibiotic use) and the outcome (intended use) were collected at the same time making it difficult to determine cause and effect, and raising concerns about reverse causality. The cross-sectional nature of our study also cannot predict future behavior without longitudinal follow-up or model development. To enhance clinical relevance, future research should focus on developing and validating a screening tool using a longitudinal study design to build a predictive model. In the meantime, the TRIPOD (Transparent Reporting of a multivariable prediction model for Individual Prognosis or Diagnosis) checklist should be applied to ensure transparent reporting. Such efforts can help inform clinical decision-making and promote safer antibiotic use. Most (65%) of our participants were interviewed over the phone due to restrictions from the COVID-19 pandemic, which prevented them from having the benefit of a visual list of commonly used antibiotics. There was also an element of social desirability and recall bias which possibly influenced the number of participants willing to admit or remember non-prescription antibiotic use. Additional studies to capture individuals in other health contexts and who do not have access to healthcare may provide different operating characteristics for such screening items. Another limitation is that this survey was done in the context of a research study and not as part of routine clinical care; the setting could affect a patient’s willingness to disclosure non-prescription antibiotic. Future research implementing such screening in clinical care is needed to confirm this screening approach and if it may indeed lead to interventions that can protect people from non-prescription antibiotic use.

## Conclusions

Succinct screening tools can identify people with the intention to use non-prescription antibiotics. A question on non-prescription antibiotic use within the last 12 months identified approximately 3 of every 4 participants who intended to use non-prescription antibiotics in the future. Those reporting non-prescription antibiotic use most often used antibiotics to treat self-limiting symptoms/illnesses. Respondents frequently took the same antibiotics they had been prescribed for similar symptoms/illnesses previously. This study proposes a method to screen for future use of non-prescription antibiotics, which may have implications on antimicrobial stewardship efforts in primary care settings.

## Data Availability

The data that support the findings of this study are not openly available due to reasons of sensitivity and privacy. Data are located in controlled access data storage at Baylor College of Medicine.

## Declarations

### Disclaimer

This work is the responsibility of the authors and does not necessarily represent the official views of the Agency for Healthcare Research and Quality or the United States Government.
